# Development of Fluorinated NP-59: A Revival of Cholesterol Use Imaging with PET

**DOI:** 10.2967/jnumed.122.263864

**Published:** 2022-12

**Authors:** Allen F. Brooks, Wade P. Winton, Jenelle Stauff, Janna Arteaga, Bradford Henderson, Jeremy Niedbala, Peter J.H. Scott, Benjamin L. Viglianti

**Affiliations:** 1 Division of Nuclear Medicine, Department of Radiology, The University of Michigan Medical School, Ann Arbor, Michigan; and; 2 The Interdepartmental Program in Medicinal Chemistry, The University of Michigan, Ann Arbor, Michigan

**Keywords:** endocrine, radiobiology/dosimetry, radiochemistry, NP-59, adrenal gland, aldosterone, cholesterol

## Abstract

Imaging of cholesterol use is possible with the ^131^I scintiscanning/SPECT agent NP-59. This agent provided a noninvasive measure of adrenal function and steroid synthesis. However, iodine isotopes resulted in poor resolution, manufacturing challenges, and high radiation dosimetry to patients that have limited their use and clinical impact. A ^18^F analog would address these shortcomings while retaining the ability to image cholesterol use. The goal of this study was to prepare and evaluate a ^18^F analog of NP-59 to serve as a PET imaging agent for functional imaging of the adrenal glands based on cholesterol use. Previous attempts to prepare such an analog of NP-59 have proven elusive. Preclinical and clinical evaluation could be performed once the new fluorine analog of NP-59 production was established. **Methods:** The recent development of a new reagent for fluorination along with an improved route to the NP-59 precursor allowed for the preparation of a fluorine analog of NP-59, FNP-59. The radiochemistry for the ^18^F-radiolabeled ^18^F-FNP-59 is described, and rodent radiation dosimetry studies and in vivo imaging in New Zealand rabbits was performed. After in vivo toxicity studies, an investigational new drug approval was obtained, and the first-in-humans images with dosimetry using the agent were acquired. **Results:** In vivo toxicity studies demonstrated that FNP-59 is safe for use at the intended dose. Biodistribution studies with ^18^F-FNP-59 demonstrated a pharmacokinetic profile similar to that of NP-59 but with decreased radiation exposure. In vivo animal images demonstrated expected uptake in tissues that use cholesterol: gallbladder, liver, and adrenal glands. In this first-in-humans study, subjects had no adverse events and images demonstrated accumulation in target tissues (liver and adrenal glands). Manipulation of uptake was also demonstrated with patients who received cosyntropin, resulting in improved uptake. **Conclusion:** ^18^F-FNP-59 provided higher resolution images, with lower radiation dose to the subjects. It has the potential to provide a noninvasive test for patients with adrenocortical diseases.

## Visual Abstract

**Figure:**
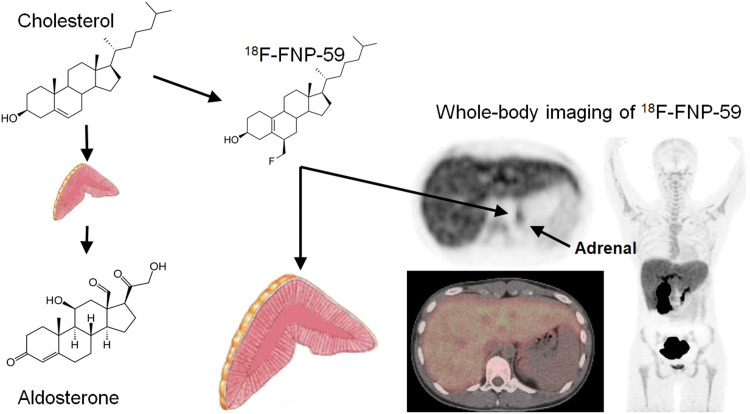

Cholesterol is essential in numerous biologic processes. Changes in the trafficking of cholesterol are an important feature of many diseases such as Cushing’s syndrome, primary aldosteronism, hyperandrogenism, adrenocortical carcinoma, and most importantly, based on number of patients affected, atherosclerosis. Given the importance of cholesterol, efforts to image its distribution and specifically its involvement in the adrenal glands was an area of focus. In the 1970s cholesterol analogs radiolabeled with ^131^I were developed as scintiscanning agents, beginning with 19-iodocholesterol (1). It was then discovered that a modification of the steroid scaffold via a thermal rearrangement gave NP-59 a remarkably improved tracer with superior adrenal uptake (2). NP-59 was subsequently developed for the use of diagnosing primary aldosteronism and other related diseases of the adrenal cortex related to the increased use of cholesterol.

The precursor for aldosterone is cholesterol, and an excessive accumulation of cholesterol esters is present in primary aldosteronism which was exploited with imaging of NP-59 to differentiate bilateral adrenal gland hyperplasia versus a unilateral solitary adenoma when blood work indicated a hormone imbalance (3–14). It has also been used to identify primary Cushing’s disease and classify an adrenal lesion as adrenal cortical carcinoma when there is a lack of uptake (2). NP-59 provided a noninvasive alternative to the gold standard for localizing abnormal cortical steroid production the invasive adrenal vein sampling procedure. However, NP-59 never saw wide adoption as a diagnostic agent given the limitations of^ 131^I: difficult synthesis, requirement of a multiday imaging protocol allowing background tissue clearance, free ^131^I accumulation in the thyroid, poor image quality due to high-energy photons emitted by ^131^I, and, consequently, poor radiation dosimetry excluding its routine screening use. Although still used in Asia and Europe in select cases, production was discontinued at our institution (the sole source in the United States) given NP-59’s limitations and subsequent improvements in CT/MRI. However, CT/MRI alone is unable to reliably differentiate bilateral adrenal gland hyperplasia versus a unilateral solitary adenoma in up to 50% of patients (15*,*^16^). In the absence of NP-59, invasive adrenal vein sampling is the only method of determining lateralization of primary aldosteronism.

Given the limitations of NP-59, many efforts to image the cause of primary aldosteronism have been investigated that do not rely on cholesterol accumulation. These include metomidate labeled with ^11^C or ^18^F that relies on the detection of extracortical adrenal tissue (17*,*^18^); fluorine-labeled ligands for CYP11B2, an enzyme involved with aldosterone production, which is overexpressed in functional adenomas (18*,*^19^); and most recently CXCR-4 ligand analogs (notably ^68^Ga-pentixafor), imaging overexpression of the receptor in adrenal adenomas (20–22). Imaging agents for these targets (CXCR-4 and CYP11B2) have the advantage of identifying adrenal adenomas based on the expression/overexpression of targets not generally seen in normal adrenal tissue, and likely have clinical utility for primary aldosteronism detection. However, as ligands they have the limitation of not being a functional imaging agent. The signal observed from their imaging does not represent the production of aldosterone or the other steroid hormones produced by the adrenal gland. Consequently, imaging agents based on the precursor (cholesterol) of aldosterone (or other cortical steroid hormones) are able to represent their production by their uptake, similar to FDG as a surrogate of glycolysis. This allows cholesterol imaging to be used for primary aldosteronism, as well as excessive cortisol production and other pathologies that rely on cholesterol.

In the years that followed the introduction of NP-59 and its study as an imaging agent for behavior as a labeled cholesterol, numerous efforts were undertaken to improve the molecule using various radionuclides and structural modifications, shown in Figure 1 (1*,*^2^*,*^23^*–*26). Prominent among these efforts was the preparation of a ^18^F analog to yield an improved PET imaging agent. However, those efforts over 3 decades failed to generate the fluorinated analog (23*,*^24^). Advances in fluorine chemistry and ^18^F radiochemistry now make the radiolabeling of a fluorinated cholesterol analog (FNP-59) possible (with results demonstrated herein). Access to ^18^F-FNP-59 will provide higher resolution images with lower radioactive dose to the subject, and potentially offer a noninvasive alternative to adrenal vein sampling.

**FIGURE 1.**
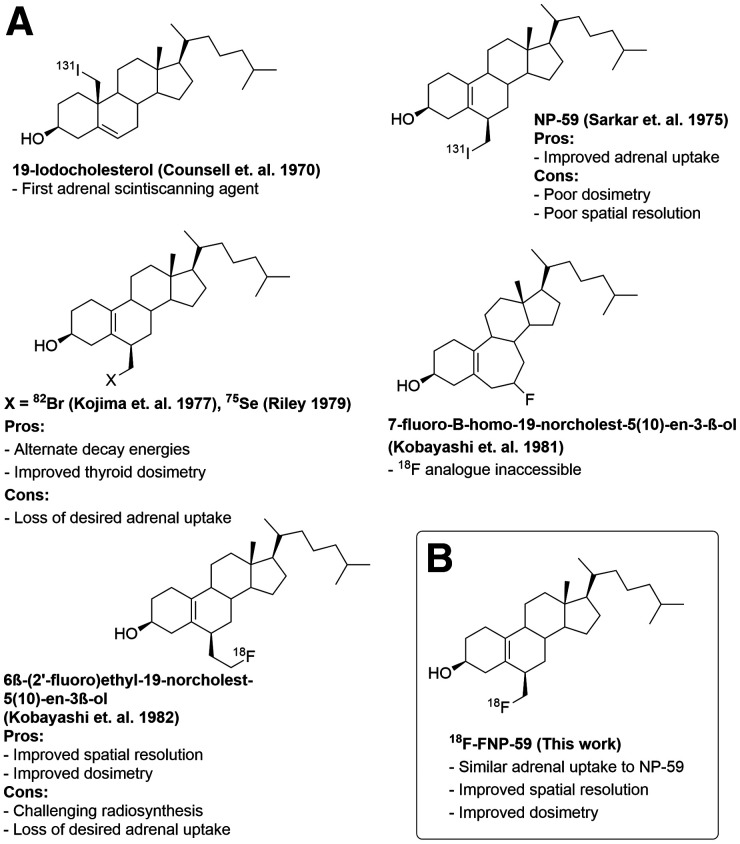
(A) Examples of previous efforts to prepare cholesterol-based adrenal imaging agents and their limitations. (B) Improved adrenal imaging agent ^18^F-FNP-59 and its advantages.

## MATERIALS AND METHODS

All animal work was done under the approval by the Institutional Animal Care and Use Committee at the University of Michigan. All human studies were performed under an Food and Drug Administration (FDA) investigational new drug (IND), registered at clinicaltrials.gov (NCT04532489 and NCT04546126), and local approval by the University of Michigan institutional review board with written informed consent obtained.

### Synthesis of FNP-59 Reference Standard and ^18^F-FNP-59

The generation of a fluorinated analog of NP-59 has been attempted for more than 40 y (24). Several advances in radiochemistry techniques have been developed by our laboratory that have allowed the development of FNP-59 (27*,*^28^). Before the synthesis of the radioactive version, a reference standard needed to be produced with nonradioactive fluoride. This reference standard would allow quality control to confirm the preparation of radioactive FNP-59 (^18^F-FNP-59) and to perform toxicity studies for evaluation and IND approval of the agent for human study.

Preparation of the reference standard for FNP-59 was achieved and published by our group (27). Once the nonradioactive FNP-59 standard was prepared, we turned our attention to ^18^F-FNP-59. We were able to achieve the synthesis using the radiofluorination chemistry techniques pioneered at our facility over the past 5 y. Starting from cholesterol (Supplemental Fig. 1; supplemental materials are available at http://jnm.snmjournals.org), this synthesis requires fewer steps and used safer chemistry techniques than the synthesis of NP-59 (2). Specifically, the radiosynthesis uses only class 3 solvents (International Conference on Harmonisation guideline for residual solvents based on solvent toxicity) and is conducted according to current good manufacturing practices (GMPs), with the resulting dose formulated at a higher specific activity than NP-59 (28*,*^29^).

Detailed synthesis procedures including quality control/high-performance liquid chromatography data for the agent and intermediate precursors are provided in the supplemental materials, or can be obtained on request from the authors.

### Preclinical Studies of ^18^F-FNP-59

#### Radiation Dosimetry

The ^18^F-FNP-59 uptake and dosimetry studies were performed in Sprague–Dawley rats (*n* = 4, 2 males/2 females) at 10, 30, 60, 120, and 360 min. Rats were anesthetized with isoflurane, and ^18^F-FNP-59 (3,349 ± 827 kBq for 10 min; 3,709 ± 255 kBq for 30 min; 3,312 ± 608 kBq for 60 min; 3,645 ± 71 kBq for 120 min; 8,297 ± 237 kBq) was administered via tail vein injection. At the appropriate time points, animals were euthanized and their tissues procured for measuring radioactivity. Radioactivity was measured in a well counter and expressed as decay-corrected percentage injected dose per gram of tissue. These data were then compared with historical ^131^I-NP-59 data as mean ± SD. Radiation dosimetry was calculated from the distribution data and was used to determine estimates of human dosimetry with OLINDA/EXM 2.0 software (29).

#### Toxicology Study

A single-dose acute toxicity study for FNP-59 was performed at the Michigan State In Vivo Facility. In this study, male and female Sprague–Dawley rats (*n* = 20, 10 males/10 females) were administered FNP-59 (416 μg/kg) intravenously at 1,000 times the expected human equivalent dose in the formulation to be used for PET imaging studies; ^18^F-FNP-59 was administered intravenously. Body weights were recorded; blood was collected at intervals for clinical chemistry and complete blood count; clinical observations were recorded daily; and food consumption was monitored. Two points (day 4 and day 15) were used for necropsies, with half of the males and females analyzed at each time point. Organs were inspected and weighed, and slides were prepared for pathology.

### In Vivo Imaging

A pilot study was performed with New Zealand rabbits. Rabbits (*n* = 2) were anesthetized with isoflurane and were dosed via intravenous administration of ^18^F-FNP-59 (83.62 ± 3.33 MBq [2.26 ± 0.09 mCi]). Imaging of the rabbits with PET (Concorde microPET) occurred at 2 and 3 h. At 4 h, the rabbits were sacrificed and imaged on a clinical PET/CT scanner (Biograph True Point; Siemens) within in 20 min of euthanasia.

### Human Imaging

With the data from the preclinical studies of ^18^F-FNP-59, the FDA approved a physician-sponsored IND (#150397; principal investigator, Benjamin L. Viglianti) in June 2020 to begin testing ^18^F-FNP-59 in human subjects. A University of Michigan Institutional Review Board (IRB) protocol (#HUM00179097) was also approved. After informed consent was obtained, 4 subjects (>18 y, no known adrenal pathology, nonpregnant females) were chosen; 2 men and 2 women were imaged dynamically for 30 min after 222 MBq (6 mCi) of ^18^F-FNP-59 were injected into the antecubital fossa. Static imaging at a 3-h time point (*n* = 4) along with a 1- and 6-h time point were also obtained (*n* = 2 for each).

An additional 3 patients without adrenal pathology were imaged under adrenal stimulation to test if we could artificially increase ^18^F-FNP-59 uptake. These patients were given 250 μg of cosyntropin intravenously over approximately 2 min. Five minutes after cosyntropin administration, 222 MBq (6 mCi) of ^18^F-FNP-59 were injected into the antecubital fossa. Dynamic imaging over the abdomen occurred for 30 min followed by a 1- and 3-h whole-body acquisition.

### Human Radiation Dosimetry and Image Analysis

Organs (liver, kidney, spleen, gallbladder, pancreas, kidneys, lungs, bones, heart, male gonads, and bladder) were segmented on MIM encore software, and SUVs, absolute, and decay-corrected/attenuated counts were generated. Given that the adrenal glands were nonenlarged, segmentation was not practical. Consequently a 2-cm sphere centered over the left adrenal gland was used for adrenal uptake measurement. The right adrenal gland was not directly measured to avoid partial-volume effect from the liver. All counts measured from the left adrenal gland were doubled to account for this.

For each patient, measured uptake data were expressed as a percentage injected dose in each organ. Time–activity curves were generated, and the resulting fractions and half-times or results of manual integrations were input into the OLINDA/EXM 2.0 software (29*,*^30^). Radiation dose to each organ or tissue was then calculated using either the ICRP Adult Male or the ICRP Adult Female models. All patients’ results were then averaged and a 95% CI for each organ was generated.

The segmentation used for radiation dosimetry was also used to report SUV versus time data. Results were reported as mean for the region of interest with a 95% confidence range.

## RESULTS

### Synthesis of FNP-59 Reference Standard and ^18^F-FNP-59

We have developed and demonstrated the synthesis of ^18^F-FNP-59 starting from cholesterol. Final products and intermediates have been confirmed via nuclear magnetic resonance spectroscopy analysis and mass spectrometry. Precursor and reference standard purity (>90%) were additionally confirmed via reversed-phase high-performance liquid chromatograph using ultraviolet detection at 212 nm; the supplemental data provides detailed data for the synthesis and characterization for standard, precursor, and intermediates. The radiosynthesis used only class 3 solvents and was conducted according to current GMPs, with the resulting dose formulated at a specific higher activity than those achieved for NP-59 (31*,*^32^).

#### Rodent Dosimetry

Biodistribution data in rats demonstrated increasing ^18^F-FNP-59 adrenal and ovary uptake over time (Supplemental Fig. 2A), as has been demonstrated to occur with ^131^I-NP-59 (2), and are consistent with the expected trafficking of cholesterol. Importantly, the adrenal-to-liver ratio is greater than 5 to 1. This result overcomes one of the main limitations of ^131^I-NP-59 imaging, the requirement of a multiday imaging protocol to allow background uptake to dissipate to resolve the image. Additionally, the data demonstrated that imaging is possible within the decay time of ^18^F (Supplemental Fig. 2C).

Radiation dosimetry estimates were calculated with OLINDA/EXM 2.0 software, using both rodent and human biodistribution data (Table 1). The results demonstrate a significantly decreased radiation dose in target organs (gonads/liver/adrenal/thyroid), compared with ^131^I-NP-59 historic data as well as an overall effective dose that is nearly 2 orders of magnitude less than ^131^I-NP-59 (29).

**TABLE 1:** Human Dosimetry for ^18^F-FNP-59 (*n* = 4; 2 Men, 2 Women), Compared with Rodent-Derived Human Estimated ^18^F-FNP-59, Human ^131^I-NP-59, and Human ^18^F-FDG

	Human sex average dose				
Target organ	mSv/MBq	±95% error, mSv/MBq	Rodent sex average dose (mSv/MBq)	Human ^131^I-NP59* average dose (mSv/MBq)	^18^F-FDG^†^ average dose (mSv/MBq)
Adrenals	2.72E–02	6.82E–03	6.82E–02	4.0E+00	1.3E–02
Brain	7.92E–03	1.41E–03	3.70E–03		1.9E–02
Breasts	8.43E–03	1.12E–02	1.17E–02	4.0E–01	9.2E–03
Esophagus	1.43E–02	1.90E–03	1.43E–02		
Eyes	7.94E–03	1.42E–03	5.22E–03		
Gallbladder wall	3.09E–01	3.73E–01	2.11E–02		1.3E–02
Left colon	1.40E–02	3.91E–03	2.85E–02		
Small intestine	1.34E–02	3.71E–03	5.14E–02	4.1E–01	1.3E–02
Stomach wall	1.48E–02	3.18E–03	1.57E–02	4.0E–01	1.3E–02
Right colon	1.88E–02	9.58E–03	5.76E–02		
Rectum	1.07E–02	2.09E–03	1.65E–02		
Heart wall	1.10E–02	3.02E–03	1.60E–02		5.9E–02
Kidneys	1.91E–02	9.46E–03	1.51E–02	4.1E–01	2.0E–02
Liver	7.29E–02	7.70E–03	3.45E–02	1.2E+00	1.6E–02
Lungs	2.19E–02	8.57E–03	2.44E–02		1.7E–02
Ovaries	1.06E–02	1.72E–02	3.11E–02	3.8E–01	1.4E–02
Pancreas	1.91E–02	1.01E–02	1.68E–02	4.3E–01	2.6E–02
Prostate	1.69E–02	1.08E–02	1.32E–02		
Salivary glands	8.90E–03	1.95E–03	1.10E–02		
Red marrow	1.07E–02	2.14E–03	1.22E–02	3.9E–01	1.3E–02
Osteogenic cells	1.66E–02	5.59E–03	1.04E–02	3.7E–01	
Spleen	5.75E–02	7.92E–02	3.36E–02	3.9E–01	3.8E–02
Testes	9.99E–03	2.94E–02	6.02E–03	3.6E–01	1.1E–02
Thymus	1.16E–02	2.16E–03	1.40E–02		1.2E–02
Thyroid	1.01E–02	2.38E–03	1.25E–02	3.0E+01	1.1E–02
Urinary bladder wall	1.11E–02	5.23E–03	1.32E–02	3.9E–01	8.6E–02
Uterus	1.03E–02	1.63E–02	1.54E–02	4.0E–01	1.7E–02
Total body	1.20E–02	2.08E–03	1.36E–02		
Effective dose	1.75E–02	5.33E–03	1.92E–02	5.6E+01	

* ICRP 53 (44).

^†^FDA product insert (45).

Significant decreased dose, 2 orders of magnitude, is seen with ^18^F-FNP-59 compared with ^131^I-NP-59.

### In Vivo Imaging Results

A pilot study in New Zealand rabbits was performed with PET images obtained at 1 and 4 h. Subsequent PET/CT images were taken immediately after euthanasia (Supplemental Fig. 3). The results demonstrate expected ^18^F-FNP-59 accumulation in the liver and the gallbladder. Gallbladder uptake was not seen in rats, as they lack a gallbladder anatomically. However, gallbladder uptake was seen in the historic NP-59 patients. More importantly, the rabbits demonstrated adrenal gland uptake in a temporal relationship similar to that in rat experiments but to a lesser degree; adrenal-to-liver ratio was approximately 2:1.

### Human Imaging Results

**FIGURE 2.**
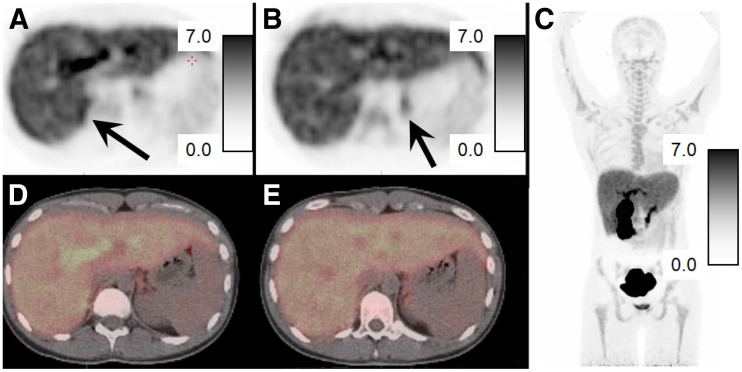
PET/CT images of ^18^F-FNP-59 in a 21-y-old woman without adrenal pathology and pretreated with cosyntropin given before 222 MBq (6 mCi) of ^18^F-FNP-59. (A and B) Axial PET images obtained at 3 h after injection of in upper abdomen, with adrenal glands (black arrows) identified on right (A) and left (B). Scale bars are 0–7 SUV. Adrenal-to-liver ratio at approximately 1.3:1 on right and 1:1.1 on left at 3 h. (C–E) Fused PET/CT images of adrenal glands are also shown (D and E) along with maximum-intensity-pixel image (C) that demonstrates expected gallbladder/biliary/bowel uptake given bile secretion.

**FIGURE 3.**
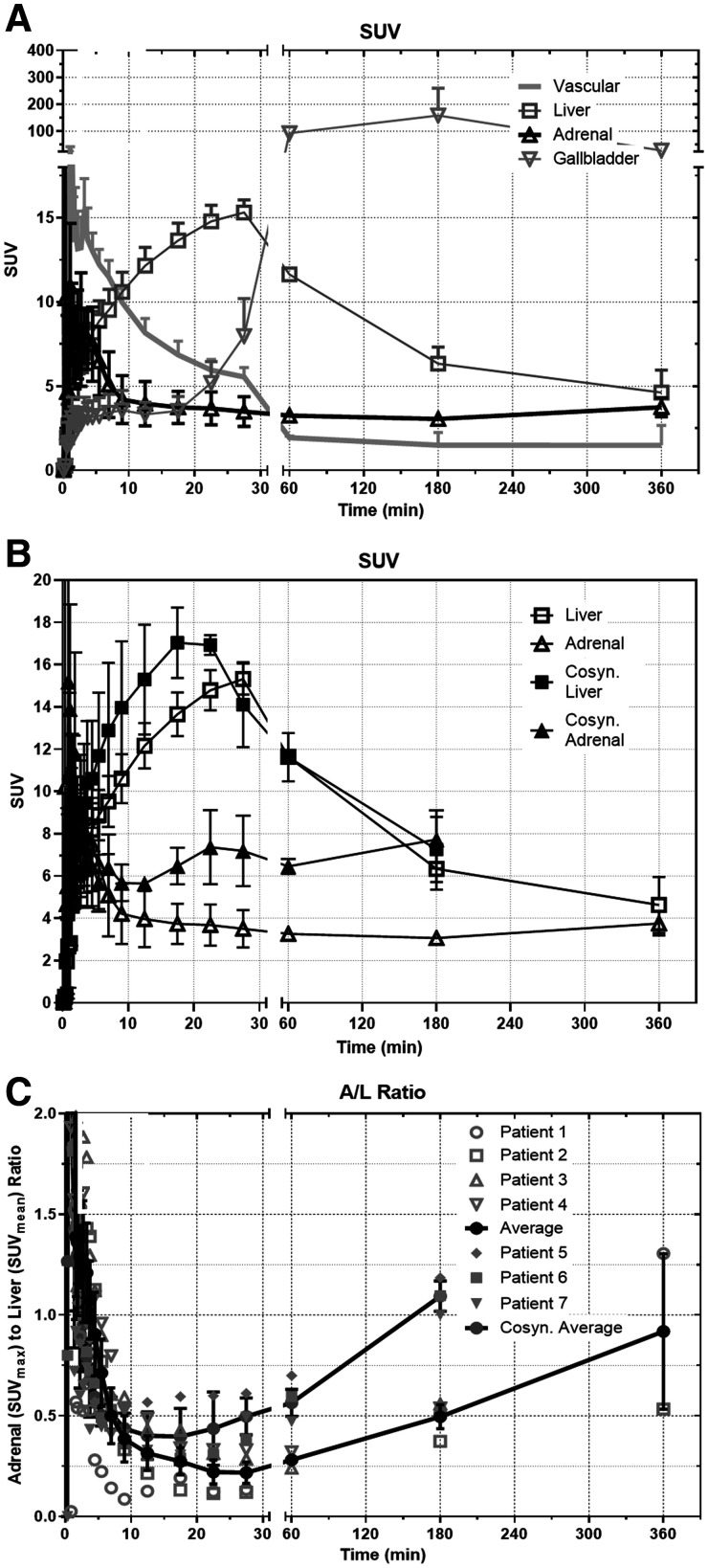
(A) SUV ^18^F-FNP-59 uptake in selected organ versus time in nonstimulated patients (*n* = 4; 2 men/2 women, at 180 min; *n* = 2 at 60 and 360 min). Over time, after first pass, liver uptake slowly clears as ^18^F-FNP-59 is excreted into bile and sent back out into enterohepatic circulation. (B) Uptake comparison in liver and adrenal gland of 3 patients stimulated with cosyntropin showing significant increased uptake in adrenal gland. Overtime, left adrenal uptake slowly increases. (C) Adrenal-to-liver (A/L) ratio is also shown, demonstrating increasing ratio over time after first pass vascular uptake (time < 30 min) is cleared. Additionally, A/L ratio of 3 female patients (5–7) given cosyntropin is shown. This cosyntropin stimulation resulted in significant increased uptake ratio compared with unstimulated patients. Error bars are SEM. *n* = 4 for dynamic imaging and at 180 min; *n* = 2 at 60 and 360 min for patients 1–4.

Four humans (2 men/2 women) were imaged with ^18^F-FNP-59. Supplemental Figure 4 and Figure 2 show examples of a 20-y-old woman and a 21-y-old woman imaged without and after an injection of cosyntropin (250 mcg), respectively. Both women had no known history of medical or endocrine disease and both were imaged at 3 h after injection of 222 MBq (6 mCi) of ^18^F-FNP-59. In all subjects, there was intense tracer uptake in the liver and gallbladder, similar to that in the rabbit experiments (Fig. 3). This uptake in the liver decreased with time as bile production occurred. Adrenal gland uptake was also seen, but was less than expected compared with the rat experiments. The adrenal to liver ratio was approximately 0.25:1 at 1 h, approximately 0.5:1 at 3 h, and about 1:1 at 6 h for an unstimulated subject (Fig. 3C), compared with animal data that suggested a 5:1 ratio at 6 h (Supplemental Fig. 2). However, this adrenal-to-liver ratio did increase over time, following kinetics similar to those in the rat experiments. Similarly, gonadal uptake (not shown) was less than the animal data had suggested it would have been.

Three subjects were pretreated with cosyntropin before ^18^F-FNP-59 administration to stimulate adrenal gland cholesterol uptake through increased hormone synthesis (Fig. 2). This pretreatment resulted in more than doubling of ^18^F-FNP-59 uptake at 1 and 3 h (Figs. 3B and 3C) along with increased uptake during dynamic phase imaging.

Overall, after injection of 40 ± 4 μg of ^18^F-FNP-59, no adverse events were observed after injection or within the following days.

## DISCUSSION

NP-59 has had a long and useful clinical history of identifying cholesterol use, accumulating in pathology where excessive production of hormones that use cholesterol as its backbone are produced. The most common use is for characterizing primary aldosteronism. Although its clinical utility has been established for approximately the past 40 y, limitations of the ^131^I label, for example, adverse dosimetry due to ^131^I, allow it to be used only in select clinical cases rather than in broader screening applications. It had been suggested that the tracer could be improved by replacing ^131^I with ^18^F (^18^F-FNP-59), but the prior chemistry techniques attempted and described in the literature were unsuccessful (23*,*^24^).

We have demonstrated an improved concise route to synthesize the FNP-59 reference standard and radiolabeling precursor. A GMP-compliant process has been developed for the production of ^18^F-FNP-59 using only class 3 solvents in accordance with green radiochemistry principles (33*,*^34^). In addition, radiation dosimetry calculations and single acute toxicity dosing studies have been conducted and showed the agent was safe and appropriate for the filing of an IND application with the FDA. The preliminary evaluation demonstrated that ^18^F-FNP-59 behaved in a manner nearly identical to historic ^131^I-NP-59 data, with a greatly improved safety profile given the 2 orders of magnitude reduction in radioactive dose to target organs, and that the required target-to-background ratios can be achieved within the physical half-life limitations of ^18^F.

In vivo imaging was performed in New Zealand rabbits. This animal model demonstrated ^18^F-FNP-59 uptake in expected tissues (adrenal glands and liver). Notably, uptake was also seen in the gallbladder of New Zealand rabbits, which was expected, and not seen in rats given their anatomic absence of a gallbladder.

First-in-humans imaging for radiation dosimetry measurements was performed in 4 individuals. There were no serious adverse events or uptake in target organs observed, and the calculated radiation dose was nearly 2 orders of magnitude less than the historic ^131^I-NP-59 radiation dose. Most important, uptake was observed in the adrenal glands, via functional imaging of cholesterol uptake. However, the adrenal-to-liver ratio was less than the animal biodistribution data would have suggested, at approximately 1:1 (Fig. 3C) rather than 5:1 (Supplemental Fig. 2A) at 6 h. Given the animal data, this ratio would likely continue to improve at later time points past 6 h. However, the physical half-life of ^18^F (109.8 min) and the sensitivity of our current equipment coupled to partial-volume effects that occur when imaging the small anatomy of a normal adrenal gland limit the ability of measuring the uptake past 6 h.

At later time points, >6 h, there would be more time available for the ^18^F-FNP-59 (acting as free cholesterol) to be incorporated into lipoproteins (primarily high-density lipoprotein) (35–37) and then accumulate in the adrenal glands through scavenger receptors while the liver and gallbladder are cleared via excretion (38). This process of cholesterol incorporation into lipoproteins and subsequent redistribution occurs much more quickly in rodents than in humans (39) and is the result of an improved adrenal-to-liver ratio seen in preclinical animal studies (Fig. 3A). This difference between rodent and humans’ redistribution was the reason that the original ^131^I-NP-59 agent was imaged 3 d after administration, which was possible given ^131^I half-life.

Although ^18^F-FNP-59 uptake in human adrenal glands was less than that observed in rodents, the data here suggest that there may be enough activity to allow imaging at time points later than the 6 h that was demonstrated. One of the proposed advantages of total-body PET/CT scanners coming online is that they offer greater sensitivity as more disintegrations are observed compared with standard equipment (40*,*^41^). However, these advantages have yet to demonstrated—a future goal we are working toward.

Although delayed imaging may demonstrate improved adrenal uptake given the rates of biologic redistribution of cholesterol, the need for delayed imaging may not be necessary when evaluating patients with pathology. Gross et al. showed that approximately 50% of normal ^131^I-NP-59 uptake in dogs (which are more similar to humans than rats in terms of how they handle cholesterol) was based on cosyntropin-stimulated cortisol production, and 10%–15% was aldosterone production (42). In a situation in which native/normal cortisol production is suppressed with dexamethasone, and a patient has pathologic primary aldosteronism, ^18^F-FNP-59 uptake may be high enough to determine laterality of abnormal production. This manipulation maneuver—dexamethasone suppression of normal cortisol production—is needed with ^131^I-NP-59 imaging to suppress normal cortisol production. When this process is performed, very minimal adrenal uptake of ^131^I-NP-59 is seen in a normal gland. A recent examination of ^131^I-NP-59 uptake in primary adrenal aldosteronism from adenoma by Lu et al. describes the adrenal-to-liver ratio of ^131^I-NP-59 as 2–2.8 after dexamethasone suppression depending on the genetic profile (43). Similarly, there was a 40%–75% increased uptake of ^131^I-NP-59 in the pathologic adrenal gland versus the normal gland. Consequently, when the data on uptake ratios and rearrangement from Lu et al. are used, the average adrenal-to-liver ratio in a normal adrenal gland for ^131^I-NP-59 would range from 1.1 to 1.6.

Stimulation with cosyntropin of 3 normal subjects demonstrated an adrenal-to-liver ratio of at least 1.2:1 at 3 h, >2 times the uptake in unstimulated subjects. If the uptake kinetics hold, the adrenal-to-liver ratio at 6 h would have been approximately 1.5–2:1. This degree of uptake is in the range of Lu et al. However, these data need to be replicated with other normal subjects stimulated with cosyntropin and imaged at later time points. More important, testing in patients who have pathology with and without dexamethasone suppression needs to be performed.

Consequently, we are currently planning to image more patients at later time points, optimizing the imaging protocol along with imaging patients with and without dexamethasone and cosyntropin stimulation. This will give us the normal expected dynamic range of uptake that can be seen with this agent. Finally, we plan to study patients who have been diagnosed with primary aldosteronism and will receive adrenal vein sampling to determine whether imaging by PET/CT using ^18^F-FNP-59 can lateralize disease.

## CONCLUSION

Overall this work demonstrates the initial feasibility of ^18^F-FNP-59 to image cholesterol trafficking and specifically uptake in human cortical adrenal tissue. Future studies will explore whether ^18^F-FNP-59 can serve as a noninvasive method to image lateral versus bilateral cause of primary aldosteronism.

## Notes

KEY POINTS **QUESTION:** Can a fluorinated NP-59 be made and successfully accumulate in the adrenal glands? **PERTINENT** **FINDINGS:** Fluorinated NP-59 was made, demonstrated improved radiation dosimetry, and accumulated in the adrenal glands. This accumulation appears dependent on a adrenal gland hormone synthesis similar to that of the prior iodine agent for which overproduction/stimulation resulted in increased accumulation. **IMPLICATIONS FOR PATIENT CARE:** With the development of FNP-59, we were able to explore whether this agent could identify the cause of primary aldosteronism (adenoma vs. bilateral hyperplasia), potentially limiting the need for adrenal vein sampling before definitive therapy.
